# Glia and Neurodevelopment: Focus on Fetal Alcohol Spectrum Disorders

**DOI:** 10.3389/fped.2014.00123

**Published:** 2014-11-11

**Authors:** Marina Guizzetti, Xiaolu Zhang, Calla Goeke, David P. Gavin

**Affiliations:** ^1^Department of Psychiatry, University of Illinois at Chicago, Chicago, IL, USA; ^2^Jesse Brown VA Medical Center, U.S. Department of Veterans Affairs, Chicago, IL, USA; ^3^Department of Environmental and Occupational Health Sciences, University of Washington, Seattle, WA, USA

**Keywords:** glia, astrocytes, oligodendrocytes, microglia, fetal alcohol spectrum disorders, neurodevelopment

## Abstract

During the last 20 years, new and exciting roles for glial cells in brain development have been described. Moreover, several recent studies implicated glial cells in the pathogenesis of neurodevelopmental disorders including Down syndrome, Fragile X syndrome, Rett Syndrome, Autism Spectrum Disorders, and Fetal Alcohol Spectrum Disorders (FASD). Abnormalities in glial cell development and proliferation and increased glial cell apoptosis contribute to the adverse effects of ethanol on the developing brain and it is becoming apparent that the effects of fetal alcohol are due, at least in part, to effects on glial cells affecting their ability to modulate neuronal development and function. The three major classes of glial cells, astrocytes, oligodendrocytes, and microglia as well as their precursors are affected by ethanol during brain development. Alterations in glial cell functions by ethanol dramatically affect neuronal development, survival, and function and ultimately impair the development of the proper brain architecture and connectivity. For instance, ethanol inhibits astrocyte-mediated neuritogenesis and oligodendrocyte development, survival and myelination; furthermore, ethanol induces microglia activation and oxidative stress leading to the exacerbation of ethanol-induced neuronal cell death. This review article describes the most significant recent findings pertaining the effects of ethanol on glial cells and their significance in the pathophysiology of FASD and other neurodevelopmental disorders.

## Introduction

Glial cells were first described by Rudolf Virchow in the middle of the nineteenth century and had been considered until recently as merely supportive and passive elements of the brain. In the last 20 years, there is accumulating evidence that rather than glial cells being supporting players in brain function, they are co-stars with neurons as new and exciting roles for them in brain development, function, and disease have emerged (1).

Neurons and macroglia (which include astrocytes, oligodendrocytes, and ependymal cells) differentiate from common precursor cells, namely neuroepithelial cells that line the cerebral ventricles and spinal canal during early brain development. Some neuroepithelial cells directly differentiate into neurons, although the majority of them are transformed into radial glial cells, which, passing through the stages of intermediate cell-type specific progenitor and precursor cells, generate neurons, oligodendrocytes, and astrocytes (2). Cell-intrinsic and -extrinsic cues regulate the differentiation of neural progenitors into each cell type, enabling the formation of functional neural circuits. Epigenetic mechanisms of chromatin remodeling appear to play a role in the cell fate specification of precursor brain cells (3). The mechanisms regulating the timing and the number of neurons and glial cells generated from a common progenitor are not fully understood. Microglia are the resident macrophages of the brain and spinal cord. The current theory regarding microglia origins is that they derive from immature erythromyeloid progenitors that migrate from the yolk sac blood islands before the blood–brain barrier starts developing (4, 5). Embryonic microglia proliferate during late gestation and early postnatal development and colonize the whole central nervous system (CNS) (4).

This review outlines the main roles played by astrocytes, oligodendrocytes, and microglia, during physiological and pathological brain development; furthermore, this review describes in detail the effects of alcohol on glial cells during brain development and highlights how these effects may contribute to the behavioral and structural effects of alcohol as seen in fetal alcohol spectrum disorders (FASD).

## Astrocytes in Brain Development

Astrocytes, the most numerous cells in the mammalian brain, have now been fully recognized as key mediators of brain development, function, and plasticity. Astrocytes serve a particularly profound role in brain development by in large part coordinating neuronal development through targeted release of trophic factors and extracellular matrix (ECM) proteins leading neurite outgrowths, allowing for neuronal survival, and controlling synapse formation and function (6–15). Furthermore, astrocytes express numerous receptors including receptors for neurotransmitters and neuromodulators that allow them to respond to cues deriving from neurons (16). Thus, astrocytes play a major role in the formation of neuronal circuits.

We demonstrated a new mechanism of astrocyte–neuron interaction by which the stimulation of astrocytes with the cholinergic agonist carbachol leads to the activation of a complex signaling involving phospholipase D (PLD), protein kinase C (PKC) ζ and ε, p70S6 kinase, NF-kB pathway, and MAPK (17–24). This results in an increase in neurite outgrowth of hippocampal neurons in astrocyte–neuron co-cultures and hippocampal slices, an effect mediated by M3 muscarinic receptors (8, 25).

Astrocytes secrete several molecules that are implicated in neurite outgrowth and synaptogenesis. We characterized astrocyte secretome by shotgun proteomic and found that most of the proteins secreted by astrocytes are involved in neuronal development and consist of ECM components as well as proteases, and protease inhibitors that modulate the levels of ECM by affecting the rate of its degradation (26). ECM proteins are indeed involved in astrocyte-modulated neuronal development processes, such as neurite outgrowth and synaptogenesis.

In recent years, several astrocyte-secreted molecules involved in the modulation of synaptogenesis have been identified including ECM proteins thrombospondin, hevin, secreted protein acidic and rich in cysteine (SPARC), membrane-anchored glypican, and lipoproteins (7, 27–29). We have reported that the stimulation of neurite outgrowth by carbachol-treated astrocytes is mediated by increased expression of the ECM proteins fibronectin and laminin in these cells and in the medium and by the upregulation of plasminogen activator inhibitor-1 (PAI-1), an inhibitor of the proteolytic degradation of the ECM (8).

Astrocytes also serve various roles in maintaining brain integrity and health. Astrocytes during brain development play a major role in the maturation, function, and maintenance of the blood–brain barrier (30, 31). In addition, together with microglia, they are responsible for immune function of the CNS contributing to the regulation of the neuroinflammatory response (32).

### Astrocytes and neurodevelopmental disorders

Recently astrocytes have been involved in the pathophysiology of several neurodevelopmental diseases. Several studies identified astrocyte dysfunction as a cause of altered development in surrounding neurons; therefore, understanding how astrocyte functions are altered in neurodevelopmental disorders is essential for the development of more effective therapeutic strategies.

Between 2009 and 2010, four independent studies were published indicating a role for astrocytes in four neurodevelopmental disorders, namely Rett syndrome, Fragile X syndrome, Fetal Alcohol Spectrum Disorders (FASD), and Down syndrome (33–36). These studies employed similar *in vitro* models of astrocyte–neuron co-cultures to demonstrate that astrocytes derived from methyl-CpG-binding protein 2 (MeCP2)-lacking mice (a model for Rett Syndrome) (33), astrocytes derived form fragile X mental retardation 1 (FMR1) knock-out mice (a model for Fragile X syndrome) (36), astrocytes pre-treated with ethanol (a model for FASD) (35), and astrocytes derived from human Down syndrome fetuses (34) foster an altered neurite and dendritic spine development in co-cultured rodent hippocampal neurons. A common finding of these studies is that, in these neurodevelopmental disorders, astrocyte dysfunction has profound effects on surrounding neurons. A follow-up study carried out in the Rett syndrome animal model showed that re-expression of MeCP2 in astrocytes improved locomotion, anxiety and respiratory patterns, prolonged lifespan, restored normal dendritic morphology, and increased the levels of the synaptic vesicle protein vGlut1 (37). Dendritic defects of FMR1-deficient neurons are significantly rescued when these cells are grown on a monolayer of wild-type rather than FMR1-deficient astrocytes. FMR1-deficient astrocytes, on the other hand, delay dendritic growth and the formation of excitatory synapses (36).

The non-cell-autonomous effects of astrocytes derived from Down syndrome fetuses on dendritic spines were attributed to reduced expression of the ECM protein thrombospondin-1 (34), which is involved in astrocyte-stimulated synapse formation (7). A recent study carried out in astrocytes and neurons differentiated from induced pluripotent cells (iPSCs) derived from Down syndrome patients demonstrated that Down syndrome astrocytes reduce neurogenesis, induce cell death, and fail to promote maturation and synaptogenesis in Down syndrome neurons. In addition, Down syndrome astrocytes display increased glial fibrillary acidic protein (GFAP) and S100B expression and nitric oxide generation, indicating that they are in a reactive state (38). The antibiotic minocycline, which has been reported to have anti-inflammatory and neuroprotective properties, partially corrects the pathological phenotype of Down syndrome astrocytes.

Reduced neuronal development has been also reported in hippocampal pyramidal neurons co-cultured with astrocytes pre-treated with developmental neurotoxicants, such as the organophosphorus insecticide diazinon, its active metabolite diazoxon, and manganese (39, 40). Alexander’s disease is a genetic disease caused by gain-of-function point mutations in the GFAP gene. Alexander’s disease is an astrogliopathy, a primary disease of astrocytes. Astrocytes are the major producers of GFAP, and Alexander disease astrocytes present Rosenthal fibers accumulation and increased levels of GFAP. Alexander’s disease is characterized by macrocephaly, abnormal white matter, and developmental delay (41, 42). The mechanism of astrocyte dysfunction is not fully understood, but the presence of white matter injury suggests effects on oligodendrocytes and myelination. Astrocyte and microglia activation caused by prenatal infections has been associated with reduced number of oligodendrocytes, altered myelination, and schizophrenia (43, 44).

In conclusion, fast-growing evidence indicates that astrocytes play a major role in neurodevelopmental disorders caused by both, genetic mutations and environmental exposures. Thus, astrocytes may be a new target for the development of therapeutic agents to treat these diseases (45, 46).

## Oligodendrocytes in Brain Development

Central nervous system myelin is an extension of the plasma membrane of oligodendrocytes wrapping multiple times around the axon where it forms a compacted sheath. Myelin allows for the process of saltatory conduction of action potentials propagated between nodes of Ranvier increasing the speed and efficiency of nerve conduction and is specific to vertebrates. Myelin, first described by Ehrenberg in 1833, was initially viewed as a static component surrounding the axons. It is now well established that the myelination process involves complex and dynamic cell–cell interactions (47, 48) that can be modified by functional experience (49, 50). In addition, there is evidence that oligodendrocytes and myelin provide trophic support to axons and promote their integrity and survival (51, 52).

Central nervous system myelination occurs late in brain development: in humans, the majority of CNS myelination occurs during the first two decades of life (53), in rodents, during the first two postnatal months. There is now evidence that in both humans and rodents, myelination continues throughout life (54, 55). Oligodendrocytes are the last cells to be generated during development, although oligodendrocyte precursor cells (OPCs) are produced much earlier in development (starting on E12.5 in mice). OPCs are produced in restricted areas and subsequently migrate and populate the entire brain where they later develop into mature oligodendrocytes. Some OPCs are present in the mature brain where they may be responsible for adult myelination (56). The myelination process is largely driven by intrinsic genetic mechanisms, but increasing evidence indicates that a major role is also played by experience-driven plasticity. Several extracellular signals, intracellular pathways, and transcription factors regulating oligodendrocyte differentiation and myelination have been characterized (52).

### Oligodendrocytes and neurodevelopmental disorders

Several types of prenatal insults have been shown to later affect myelination, indicating that effects on the generation or survival of OPCs during fetal development are responsible for altered myelin in the adult brain. Indeed, it has been reported that gestational exposure to stresses, including hypoxia, restraint stress, opioids, vitamin B12 deficiency, and methamphetamine decrease myelination in the offspring postnatal brain (57–61). Prenatal infections associated with astrocyte and microglia activation induce myelin and oligodendrocytes abnormalities and may be linked to schizophrenia (43, 44).

## Microglia in Brain Development

Microglia are resident CNS immune cells that represent approximately 10% of the total brain cell population (62). Microglia are of hematopoietic origin; their main function in the adult brain is to monitor the environment and to respond to infection and injury. Microglia exist in two conformations: surveying microglia, presenting a ramified morphology, and activated microglia, which can assume an ameboid, rod, multinucleated, or epithelioid morphology (62). Microglia respond to almost all types of CNS insult by switching from a surveillance state to one of the activated states that involves changes in cell morphology, gene expression, and function (62). Activated microglia produce many pro-inflammatory mediators, including cytokines, chemokines, reactive oxygen species (ROS), and nitric oxide. Furthermore, microglia can become phagocytic and contribute to the clearance of pathogen infections and toxic cellular debris after injury. While microglia activation is aimed at protecting neurons from infections, it can also trigger extensive and damaging neuroinflammation that contribute to the progression of neurodegenerative diseases (63).

Microglia are present in the amoeboid morphology during embryonic development and transition toward a ramified morphology during early postnatal development in rodents. During CNS development, microglia play a major role in the refinement of brain wiring and synaptic circuits (64–66). Neuronal apoptosis and synaptic pruning are important physiological processes occurring in the developing brain where more than the necessary neurons and synapses are generated. In the immature brain, amoeboid microglia have an active role in phagocytosis of apoptotic neurons, promotion of programmed cell death (67, 68), and pruning of synapses (69). Mechanisms inducing engulfment of synaptic structures and synaptic pruning by microglia include activity-dependent mechanisms (70), fractalkine signaling (69), and modulation of complement protein release by developing synapses (71).

### Microglia and neurodevelopmental disorders

Several lines of evidence implicate microglia in autistic spectrum disorders (ASD) (72). Several markers of neuroinflammation have been reported in the brain of ASD patients. Indeed, high levels of cytokines, which are expressed in the brain by activated microglia (73), are found in the brains of autistic patients (74, 75). Furthermore, microglial activation has also been demonstrated in several brain regions of ASD patients (76–78).

In a mouse model of Rett syndrome, a genetic disease of the ASD spectrum caused by mutations in the MeCP2 protein, microglia display reduced phagocytic activity; the motor abnormalities present in MeCP2 knock-out animals are partially rescued by replacing MeCP2 in microglia (79). MeCP2-null microglial cells release glutamate, delay neuronal development, and trigger neurotoxicity in hippocampal neurons (80).

Epidemiologic studies show that prenatal exposure to infections is associated with increased risk of adult schizophrenia (81). Because microglia are the main player in neuroinflammatory responses, microglia may be implicated in alterations in brain circuits during the development caused by prenatal infections, which may be involved in schizophrenia (43, 44).

## Fetal Alcohol Spectrum Disorders

FASD are a heterogeneous group of conditions defined as the physical, behavioral, and learning impairments that occur in the offspring of women who drank alcohol during pregnancy (82, 83). FASD include fetal alcohol syndrome (FAS), partial FAS (pFAS); alcohol-related neurodevelopmental disorders (ARND), and alcohol-related birth defects (ARBD) (84, 85). Neurobehavioral deficits associated with heavy prenatal alcohol exposure include reduced IQ and impairments in several neurodevelopmental domains such as attention, reaction time, visuospatial abilities, executive functions, fine and gross motor skills, memory, language, and social and adaptive functions (86).

Glia involvement in FASD is suggested by the fact that the brains of individuals with FASD present with abnormal glial migration (87) and hypoplasia of the corpus callosum and anterior commissure, two areas originally formed by neuroglial cells (88). Furthermore, the finding that microencephaly is strongly associated with ethanol exposure during the brain growth spurt (89), a period characterized by rapid glial cell proliferation and maturation, also suggests a potential effect of ethanol on the proliferation, growth, and maturation of glia. In experimental models, the involvement of glial cells in the developmental effects of ethanol has been recognized for more than 20 years. Earlier findings pertaining to glia and FASD have been summarized in an excellent review (90); therefore, the present review will focus mostly on research published during the last 15 years.

## Astrocytes and FASD

### Astrocyte proliferation in FASD

Several early studies investigated the effects of ethanol on astrocyte proliferation. The incubation of primary astrocytes in culture with ethanol inhibits astrocyte proliferation induced by serum, M3 muscarinic receptor stimulation, and IGF-1 (91–93). Subsequent studies revealed selectivity in the signaling pathways affected by ethanol. Muscarinic receptor-mediated activation of phospholipase C, subsequent increase in intracellular calcium, and activation of novel PKCε and of mitogen-activated protein kinases are relatively unaffected by ethanol (24, 94). On the other hand, PLD-mediated formation of phosphatidic acid and sequential activation of atypical PKCζ, p70S6 kinase, and of NF-κB are all strongly inhibited by ethanol (17, 20, 22).

**Figure 1:**
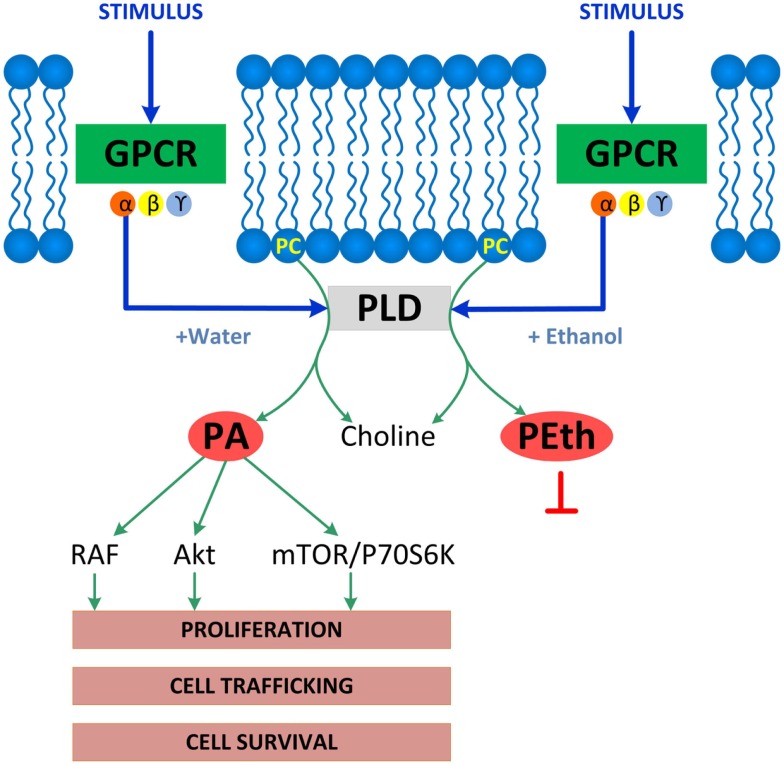
**Enzymatic reactions catalyzed by PLD**. PLD is associated with membrane receptors including G-protein coupled receptors (GPCR), receptor tyrosine kinases, or integrins, which all activate PLD. Shown is GPCR-coupled PLD, which, upon activation under physiological conditions, hydrolyzes phosphatidylcholine (PC) to produce choline and phosphatidic acid (PA), a lipid second messenger that binds and activates several signaling molecules including RAF, Akt, mTOR, and p70S6K and stimulates several cell functions including proliferation, cell trafficking, and cell survival. The PLD signaling pathway is disrupted by ethanol, which competes with water leading to the formation of phosphatidylethanol (PEth) at the expenses of phosphatidic acid, therefore, inhibiting phosphatidic acid-activated signaling and functions.

Phospholipase D-induced phosphatidic acid formation was identified by two groups as the direct target of ethanol in the inhibition of muscarinic receptor- and serum-stimulated signaling in astrocytes (22, 95). Indeed, ethanol is a competitive substrate for PLD leading to the formation of phosphatidylethanol instead of the physiological second messenger phosphatidic acid (96). Phosphatidic acid activates a myriad of signaling molecules, including RAF, mTOR, p70S6K, and Akt and is involved in several cellular functions, including intracellular trafficking, survival, and proliferation (97, 98) (Figure 1). Inhibition of astrocyte proliferation by ethanol is consistent with the reduced number of glial cells found following *in vivo* ethanol exposure (99, 100), and may contribute to ethanol-induced microencephaly (89).

### Astrocytes and neuronal plasticity in FASD

Several paradigms of neuronal plasticity are altered by alcohol exposure during brain development in animal models of FASD, as recently summarized in an excellent review article (101). A large body of evidence suggests that structural plasticity is highly affected by *in utero* alcohol exposure. Indeed, specific cortical maps are altered in FASD models (102–104). Furthermore, prenatal and/or neonatal alcohol exposure reduces dendritic branching and dendritic spine density in hippocampal and neocortical pyramidal neurons (105–110).

The importance of astrocytes in ethanol-induced changes in neuritogenesis is indicated by co-culture experiments. Medium from ethanol-cultured astrocytes was shown to impair neuronal survival and neuritogenesis of rhombencephalic serotoninergic neurons in culture (111). Astrocytes have also been reported to modulate the effect of ethanol on dendritic development with different outcomes depending on the time of ethanol exposure (112). Further, neuritogenesis is inhibited in cortical neurons grown in the presence of astrocytes prepared from rats prenatally exposed to ethanol in comparison to neurons incubated with astrocytes from unexposed animals (113).

Several factors released by astrocytes have been implicated in the effects of ethanol on neuritogenesis. The active fragment of the astrocyte-released activity-dependent neuroprotective protein (ADNP) has been implicated in ethanol’s effects on axonal growth in cerebellar neurons (114). Serum response factor (SRF) in astrocytes has also been suggested to play a role in the effects of ethanol on neuritogenesis. Indeed, overexpression of SRF in astrocytes restores ocular dominance plasticity in a ferret model of FASD (115, 116).

**Figure 2:**
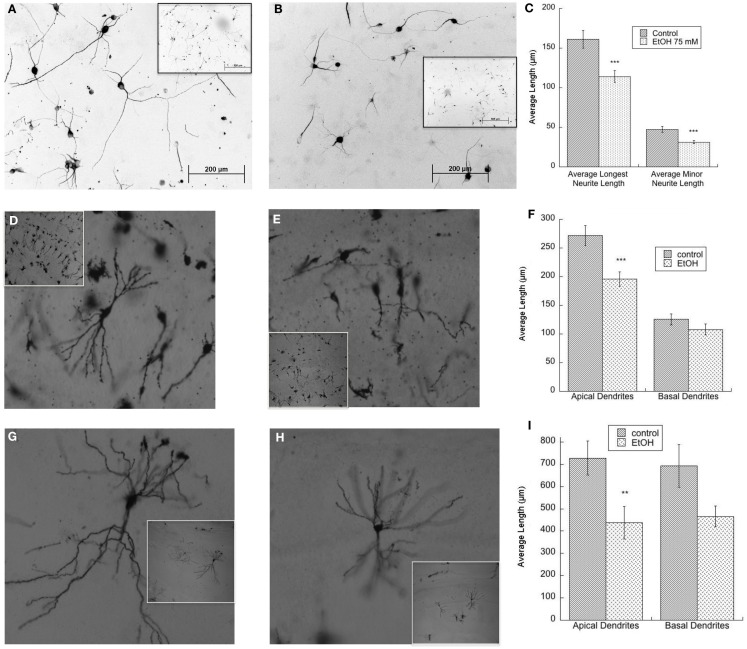
**(A–C)** Ethanol-treated astrocytes inhibits hippocampal neuron neurite outgrowth. Hippocampal neurons plated on top of ethanol-pre-treated astrocytes (75 mM) display reduced neurite outgrowth. Shown are representative fields (20×) of neurons incubated with control **(A)** and ethanol-treated **(B)** astrocytes; *insets* show the same fields at a lower magnification (10×). **(C)** Quantification of the length of the longest neurite and of minor neurite in 60 cells per treatment was carried out using the software Image J. ***p < 0.001, Student’s *t* test. **(D–F)** Neonatal ethanol exposure inhibits dendrite outgrowth in PD9 rats. Male rat pups were intubated with 5 g/kg ethanol or were sham (control) intubated from PD4 to PD9 and sacrificed on PD9. The brains were stained using the Golgi-Cox procedure. Representative CA1 neurons in control **(D)** and ethanol-exposed rats **(E)** are shown (10×); *insets* show the same fields at a lower magnification (4×). Dendrite length was measured using the software Neurolucida **(F)**. ***p < 0.001 by Student’s *t* test. **(G–I)** Neonatal ethanol exposure reduces dendrite length in PD36 rats. Female rat pups were intubated with 5 g/kg/day of ethanol or were sham (control) intubated from PD4 to PD9 and sacrificed on PD36. Brains were stained using the Golgi-Cox procedure. Shown are representative CA1 hippocampal neurons in control **(G)** and ethanol-exposed rats **(H)** (10×); *insets* show the same fields at a lower magnification (4×). Dendrite length was measured using the software Neurolucida **(I)**. **p < 0.01, Student’s *t* test.

We have reported on the important role of muscarinic receptors in ethanol-induced impairment in neuritogenesis related to FASD. Hippocampal neuron neuritogenesis stimulated by astrocyte muscarinic receptor is inhibited by physiologically relevant concentrations of ethanol in an astrocyte–neuron co-culture model in which the two cell types are not in direct contact, an effect confirmed also in hippocampal slices (25, 35). In addition, in the absence of muscarinic receptor stimulation, ethanol-treated astrocytes displayed a reduced ability to foster neurite outgrowth when hippocampal pyramidal neurons and astrocytes are in direct contact (117) (Figures 2A–C). Reduced pyramidal neuron development is also observed in the hippocampus of postnatal day (PD) 9 and PD 36 rats exposed to ethanol between PD 4 and PD 9, a model of alcohol exposure mimicking the third trimester of human exposure (Figures 2D–I), in agreement with what reported by others in hippocampal and cortical pyramidal neurons (106, 109).

Ethanol inhibits astrocyte-mediated neurite outgrowth by profoundly affecting astrocyte secretion leading to the generation of an environment that is repressive of neuronal development. We have characterized several mechanisms by which ethanol affects astrocyte secretion:

(a) Ethanol reduces the release of neuritogenic ECM proteins laminin and fibronectin stimulated by muscarinic receptors in astrocytes (35), which may be due to the perturbation of the secretory pathway induced by ethanol in astrocytes (118).
(b) Ethanol induces a dysregulation of the plasminogen activator system, an important player in ECM proteolysis. Plasminogen is converted to the active proteolytic enzyme plasmin by two plasminogen activators: tissue plasminogen activator (tPA) and urokinase plasminogen activator (uPA), which are regulated by plasminogen activator inhibitor-1 (PAI-1), a member of the serine protease inhibitor superfamily. PAI-1 binds and inhibits plasminogen activators, thereby preventing the proteolytic activation of plasminogen to plasmin and the degradation of the ECM (119). We observed that ethanol inhibits the upregulation of PAI-1 induced by muscarinic receptor activation, therefore, increasing the degradation of EMC proteins laminin and fibronectin. The inhibitory effect of ethanol is in part mediated by ethanol-induced inhibition of the PLD signaling in astrocytes (Figure 1) (35).
(c) In addition, we found that ethanol increased the levels and release of tPA in astrocytes (120). Tissue-PA is upregulated by alcohol in the brain of animal models of both alcoholism and FASD, where it reduces the levels of laminin and causes neurodegeneration (121, 122). We found that the upregulation of tPA by ethanol is mediated by the epigenetic mechanism of DNA methylation. Indeed, ethanol inhibits DNMT activity and DNA methylation in the promoter region of tPA, thereby increasing tPA expression in astrocytes (120).
(d) Ethanol through the inhibition of arylsulfatase B (ARSB) activity increases the levels of chondroitin-4-sulfate and of the chondroitin sulfate proteoglycan neurocan, which are inhibitors of neurite outgrowth, in astrocytes *in vitro* and after neonatal alcohol exposure *in vivo* (117).

Together, the evidence discussed in this section underscores the role played by astrocytes in neuronal structural plasticity during brain development and outlines the novel and very important mechanism by which ethanol affects neuronal plasticity through alterations in astrocyte secretion.

### Astrocytes, oxidative stress, and FASD

Astrocytes are immunoresponsive cells. Several *in vitro* studies reported that ethanol induces oxidative stress in astrocytes in culture. Indeed, ethanol stimulates the formation of ROS, depletes glutathione, and upregulates cyclooxygenase 2 and inducible nitric oxide synthase expression via NF-κB activation in astrocytes (123, 124).

Ethanol activates Toll-like receptor 4/interleukin 1 receptor 1(TLR4/IL-1R1) signaling in astrocytes; inhibition of TLR4 and IL-1R1 abolishes ethanol-induced NF-κB and AP-1 activation, inducible nitric oxide synthase, and cyclooxygenase-2 upregulation indicating that these receptors mediate ethanol-induced inflammatory events in astrocytes (125). On the other hand, cortical neurons are more sensitive than astrocytes to ethanol and undergo apoptotic cell death mediated by increased ROS production and GSH depletion; attenuated neuronal cell death and reduced GSH depletion was observed when neurons are co-cultured with astrocytes (126, 127).

### Astrocytes and brain lipid homeostasis in FASD

The maintenance of optimal cholesterol levels is essential to brain development. As in the periphery, cholesterol is circulated in the brain associated with lipoproteins, which are produced by astrocytes; astrocytes and microglia, but not neurons, also express apolipoprotein E (apo E). Lipoproteins produced and released by astrocytes are discoidal in shape and contain apo E, phospholipids, and cholesterol, but lack in the core lipids (cholesterol esters or triglycerides). In contrast, lipoproteins found in the cerebrospinal fluid are round, contain a cholesterol ester core, and are similar to plasma high-density lipoproteins (HDL) (128, 129). An important mechanism of brain cholesterol clearance involves cholesterol efflux from brain cells to astrocyte-released lipoproteins, which exit the brain after passing from the brain parenchyma into the cerebrospinal fluid and across the blood–brain barrier (130–132).

We have been investigating the hypothesis that ethanol increases lipoprotein release from astrocytes leading to increased cholesterol clearance and reduced levels of cholesterol in the whole brain (133). The transporter ABCA1 (ATP-binding cassette-A1) is essential for the generation of nascent lipoproteins in astrocytes, subsequent cholesterol efflux is mediated by ABCG1 and ABCG4 transporters and lead to the lipidation and remodeling of nascent, lipid-poor lipoproteins (134–136).

**Figure 3:**
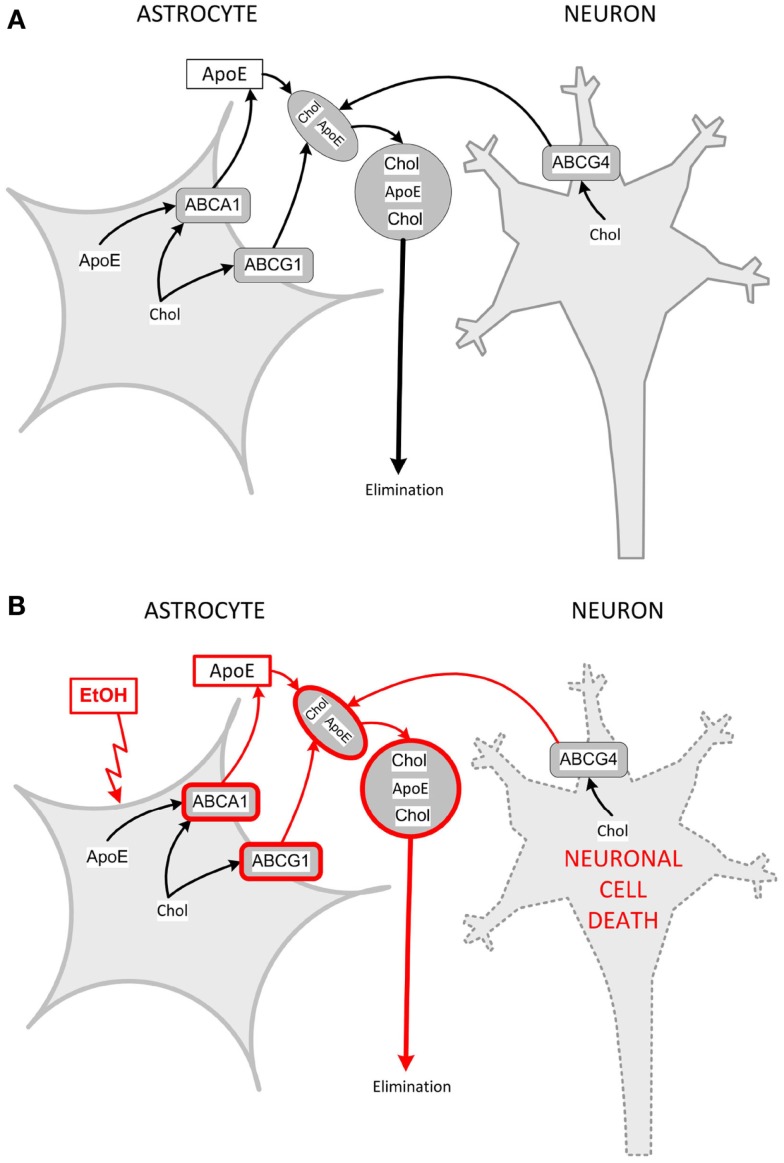
**Model for interactions of astrocytes and neurons in cholesterol clearance and dysregulation by ethanol**. **(A)** Cholesterol is produced by both astrocytes and neurons. Astrocytes produce and release nascent, lipid-poor lipoproteins through the lipidation of apoE via the membrane-bound ABCA1. In the brain parenchyma, nascent lipoproteins stimulate cholesterol efflux through their interaction with ABCA1 and ABCG1 in astrocytes and ABCG4 in neurons. Lipoprotein-associated cholesterol exits the brain through the cerebrospinal fluid. **(B)** Ethanol upregulates ABCA1 and ABCG1 in astrocytes, and increases the formation of astrocyte lipoproteins, which extract more cholesterol from astrocytes and neurons (pathways upregulated by ethanol are in red). Neurons are sensitive to changes in cholesterol content and, upon protracted induction of cholesterol efflux, decrease their cholesterol content and undergo cell death. Chol, cholesterol.

We have shown that ethanol increases ABCA1 and ABCG1 levels, induces cholesterol efflux, and reduces cholesterol levels in primary rat astrocytes in culture (137). Interestingly, isotretinoin, which causes developmental effects similar to ethanol, induces ABCA1 and ABCG1 expression, increases cholesterol efflux, and decreases cholesterol content in astrocytes similarly to ethanol (137, 138), suggesting a common mechanism of teratogenesis (133). These observations were also confirmed in *in vivo* FASD models. Indeed, neonatal alcohol exposure increases cortical levels of ABCA1 (137); furthermore, prenatal alcohol exposure upregulates ABCA1 and ABCG1 and reduces the levels of cholesterol in the neocortex of GD 21 female fetuses (139). Figure 3 shows a proposed model of the interactions of astrocytes and neurons in cholesterol homeostasis and the effects of ethanol.

### Astrocyte differentiation in FASD

Published literature support the notion that the survival of progenitor cells and their differentiation into astrocytes is inhibited by ethanol leading to an overall decreased astrocyte population, which may strongly affect neuronal development and survival given the trophic role played by astrocytes.

Ethanol reduces the survival of neural progenitors from human embryonic stem cells and their differentiation into astrocytes (140, 141). Furthermore, ethanol exposure during embryogenesis reduces the telencephalic radial glia progenitor pool and its differentiation into neurons and astrocytes (142). Ethanol inhibits precursor cell proliferation and astrogliogenesis also in a rodent neurosphere culture model (143).

## Oligodendrocytes and FASD

Most of the earlier studies on the effects of fetal alcohol on myelination and oligodendrocytes have been carried out between the late 1970s and the early 1990s followed by a paucity of papers emerging for more than a decade. However, the last few years were characterized by a resurrected interest in the effects of alcohol on myelination during brain development. This was triggered by advancements in imaging techniques allowing for better evaluation of white matter damage in FASD individuals.

Oligodendrocyte differentiation and myelination in humans occur mostly after birth; however, myelination is affected by *in utero* alcohol exposure indicating that fetal alcohol effects on OPCs results in altered oligodendrocyte development and myelination. Indeed, imaging studies have found global white matter reduction and white matter abnormalities in children and adolescents with FASDs (144, 145). Myelination is affected in several *in vivo* FASD models. In developing rat brains, exposure to ethanol alters myelin ultrastructure and delays myelination (146–148). It was more recently reported that myelin is disrupted and oligodendrocyte morphology is altered also in a third-trimester equivalent sheep model of FASD (149).

While abnormalities and delays in the biochemical profile of myelin (150–152) and in myelin protein synthesis were reported following prenatal alcohol exposure in rats, more dramatic effects were obtained after ethanol exposure during the third-trimester equivalent (i.e., the first 10 PDs), when oligodendrocytes begin maturation and myelination (148). Several studies report delayed or reduced expression of oligodendrocyte proteins suggesting delayed differentiation. Ethanol delays the expression of myelin basic protein (MBP) and the maturation of oligodendrocytes cultured from PD 1–2 rats prenatally exposed to ethanol (153). Postnatal ethanol exposure reduces the levels of MBP and myelin-associated glycoprotein also in the cerebellum of PD15 rats (154). Direct exposure of oligodendrocytes in culture to ethanol confirms an inhibited expression of MPB (155). Recently, widespread oligodendrocyte apoptosis has been reported in the white matter regions of the fetal brain of monkeys exposed to high alcohol levels during the equivalent of the third trimester of human gestation (156).

Hypoplasia of the optic nerve is common in individuals with FAS and may be responsible for their reduced visual function (157). Several alterations in the optic nerve myelin have been reported in animal models of FASD, including permanent reduction in myelin thickness, fewer myelinated axons, aberrant myelin sheaths, and myelin acquisition, which may account for fetal alcohol-induced hypoplasia of the optic nerve (147, 158–160).

Together, these studies clearly indicate that oligodendrocytes development and survival are affected by fetal alcohol leading to altered myelination, which may have a great impact on axonal size and ability to effectively transmit action potentials. Further research is required to fully understand the mechanisms involved in the effects of prenatal ethanol on oligodendrocyte development and myelination.

## Microglia and FASD

Microglial cells can be directly activated by alcohol. Indeed, ethanol, by activating TLR2 and TLR4 signaling in microglia, triggers phagocytosis and production of ROS and cytokines, factors that contributes to inflammation and cortical neuron apoptosis (161, 162). Microglia play also a role in alcohol-induced apoptosis of developing hypothalamic neurons. Indeed, ethanol increases the release of inflammatory cytokines form microglia and induces oxidative stress and decreases the intracellular levels of cAMP and BDNF in hypothalamic neurons co-cultured with ethanol-treated microglia (163–165); Ethanol also induces microglia cell death both *in vitro* and after neonatal alcohol *exposure in vivo*, effects that are prevented by a PPRγ agonist (166). The effects of alcohol on neuroinflammation processes involving microglia activation in fetal, adolescent, and adult brain are the topic of several recent review articles and book chapters (167–170).

## Concluding Remarks and Future Directions

Recent studies indicate the essential role glial cells play in the developing brain. For example, growth factors released by astrocytes guide neuritogenesis and support neuronal survival, microglia mediate synaptic pruning, and oligodendrocytes regulate axonal size, function, and survival. In this way, the development of brain circuits depends on glia–glia and glia–neuron interactions. In addition, several mechanisms underlying many neurodegenerative and neurodevelopmental diseases are non-cell-autonomous and involve glia–neuron interactions (171).

**Figure 4:**
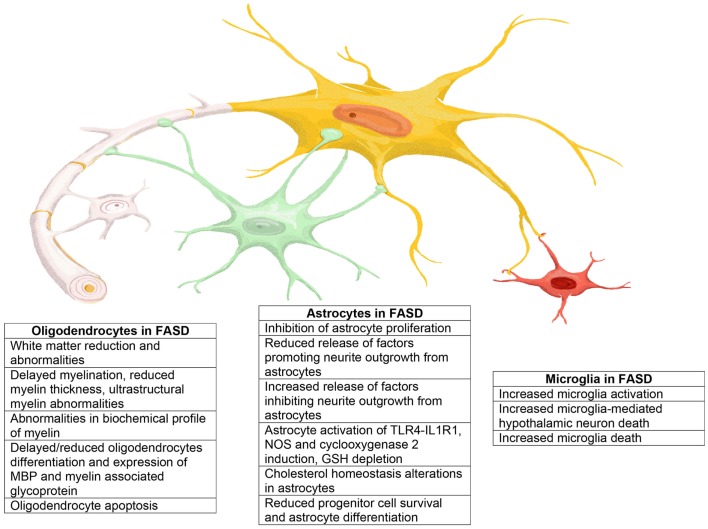
**Representation of the main effects exerted by ethanol on astrocytes, oligodendrocytes, and microglia that may play a role in the neuropathology of FASD**.

In this review, we summarize published evidence that glia–neuron interactions play an essential role also in the pathophysiology of FASD. Astrocytes, oligodendrocytes, and microglia are all highly affected by *in utero* alcohol exposure as summarized in Figure 4. The impact of alcohol exposure on the brain should not be considered as the sum of the isolated action of ethanol on neurons, astrocytes, oligodendrocytes, and microglia. Indeed, it is clear that alcohol not only affects signaling pathways within a cell type but also strongly alters the ability of glial types to send information to neurons and *vice versa*, therefore, greatly disrupting the synchronized series of events that lead to the correct development of the brain and the proper formation of the brain circuit architecture. Thus, it is imperative that novel therapeutic strategies for the treatment or prevention of the neurodevelopmental effects of ethanol target not only the functions of individual cell types but also mechanisms of glia–glia and glia–neuron communications.

## Conflict of Interest Statement

The authors declare that the research was conducted in the absence of any commercial or financial relationships that could be construed as a potential conflict of interest.

## Abbreviations

ABCA1, ATP-binding cassette-A1; ABCG1, ATP-binding cassette G1; ADNP, activity-dependent neuroprotective protein; ARSB, arylsulfatase B; ASD, autism spectrum disorders; CNS, central nervous system; ECM, extracellular matrix; FASD, fetal alcohol spectrum disorders; FMR1, fragile X mental retardation 1; IL1-R1, interleukin1-receptor 1; iPSCs, induced pluripotent cells; MBP, myelin basic protein; MeCP-2, methyl-CpG–binding protein 2; OPCs, oligodendrocyte precursor cells; PAI-1, plasminogen activator inhibitor-1; PD, postnatal day; PKC, protein kinase C; PLD, phospholipase D; SPARC, secreted protein acidic and rich in cysteine; SRF, serum response factor; TLR, toll-like receptor; tPA, tissue plasminogen activator; uPA, urokinase plasminogen activator.
