# Corrigendum: “Glia and Neurodevelopment: Focus on Fetal Alcohol Spectrum Disorders”

**DOI:** 10.3389/fped.2015.00027

**Published:** 2015-04-21

**Authors:** Marina Guizzetti, Xiaolu Zhang, Calla Goeke, David P. Gavin

**Affiliations:** ^1^Department of Psychiatry, University of Illinois at Chicago, Chicago, IL, USA; ^2^Jesse Brown VA Medical Center, U.S. Department of Veterans Affairs, Chicago, IL, USA; ^3^Department of Environmental and Occupational Health Sciences, University of Washington, Seattle, WA, USA

**Keywords:** fetal alcohol spectrum disorders, glia, astrocytes, microglia, oligodendrocytes

During the preparation of the paper the authors neglected to list all funders, the following funders were not included in the original version: Department of Veterans Affairs Merit Review Awards #I01BX001819 (MG) and Career Development Award (CDA-2) #IK2BX001650 (DG). The complete Acknowledgement section should read.

This study was supported in part by grant AA021876 from the National Institute of Alcoholism and Alcohol Abuse, Department of Veterans Affairs Merit Review Awards #I01BX001819 (MG) and Career Development Award (CDA-2) #IK2BX001650 (DG). The authors are extremely grateful to Mr. Jeff Frkonja for the graphic design of Figures 1, 3, and 4.

The authors would like to apologize for any potential inconvenience caused.

## Conflict of Interest Statement

The authors declare that the research was conducted in the absence of any commercial or financial relationships that could be construed as a potential conflict of interest.

